# Astrocytes as critical players of the fine balance between inhibition and excitation in the brain: spreading depolarization as a mechanism to curb epileptic activity

**DOI:** 10.3389/fnetp.2024.1360297

**Published:** 2024-02-09

**Authors:** Rune Enger, Kjell Heuser

**Affiliations:** ^1^ Letten Centre and GliaLab, Division of Anatomy, Department of Molecular Medicine, Institute of Basic Medical Sciences, University of Oslo, Oslo, Norway; ^2^ Department of Neurology, Oslo University Hospital, Rikshospitalet, Oslo, Norway

**Keywords:** astrocyte, spreading depression, spreading depolarization, epilepsy, migraine, seizure termination

## Abstract

Spreading depolarizations (SD) are slow waves of complete depolarization of brain tissue followed by neuronal silencing that may play a role in seizure termination. Even though SD was first discovered in the context of epilepsy research, the link between SD and epileptic activity remains understudied. Both seizures and SD share fundamental pathophysiological features, and recent evidence highlights the frequent occurrence of SD in experimental seizure models. Human data on co-occurring seizures and SD are limited but suggestive. This mini-review addresses possible roles of SD during epileptiform activity, shedding light on SD as a potential mechanism for terminating epileptiform activity. A common denominator for many forms of epilepsy is reactive astrogliosis, a process characterized by morphological and functional changes to astrocytes. Data suggest that SD mechanisms are potentially perturbed in reactive astrogliosis and we propose that this may affect seizure pathophysiology.

## Introduction

Epilepsy is one of the most common neurological disorders—estimated to affect 65 million people worldwide (Hesdorffer et al., 2011; Neligan et al., 2012; Beghi, 2016). It is a chronic disorder, characterized by sudden, violent perturbations of normal brain functions, accounting for much stigma, morbidity and in some cases death for the affected individuals. There is a striking lack of knowledge of the specific cellular mechanisms at play in epilepsy. For instance, the process transforming normal brain matter to a focus for epileptic seizures is elusive. Furthermore, key questions like “*What cellular mechanisms set in motion an epileptic seizure?*” and “*What terminates seizure activity?*” remain unanswered. Failure of the mechanisms that curb or prevent hyperexcitation have been increasingly recognized as probable key pathogenic factors of epilepsy.

Spreading depolarizations (SD) are slow waves of complete depolarization of gray matter followed by neuronal silencing, involved in a range of brain disorders. During SD profound changes occur in the brain tissue. For instance, transient hypoxia, cellular swelling, a temporary breakdown of the blood-brain barrier, as well as severely perturbed transmembrane ionic gradients are known to occur (Takano and Nedergaard, 2009; Charles and Baca, 2013). SD was first discovered in 1944 by Aristides Leão while studying seizure activity in rabbits. He discovered a wave-like phenomenon of silencing of brain function that occurred in the seizing brain (Leao, 1943). Since then a large body of literature addresses the role of SD in brain disorders (Dreier, 2011; Lauritzen et al., 2011; Ayata and Lauritzen, 2015). The phenomenon is believed to be the cellular substrate of the migraine aura, and in ischemia, brain trauma and subarachnoid hemorrhage, SD is believed to add insult to injury by increasing the metabolic demand of an already compromised tissue (Takano and Nedergaard, 2009; Dreier, 2011; Lauritzen et al., 2011; Charles and Baca, 2013; Hartings et al., 2020; Dreier et al., 2022). While the role of SD in for instance ischemia and migraine has been quite extensively explored, the role for SD in epilepsy and seizures is much less studied. Seizures and SD are both paroxysmal hyperexcitability phenomena that share elemental pathophysiological features (Rogawski, 2008; Dreier et al., 2012; Wei et al., 2014; Mantegazza and Cestèle, 2018). Importantly recent evidence clearly indicates that SD is a frequent occurrence in a range of acute and chronic experimental seizure models (Aiba and Noebels, 2015; Khoshkhoo et al., 2017; Heuser et al., 2018; Bahari et al., 2020; Tamim et al., 2021). Data from humans are scarce, although a few studies have demonstrated co-occurring seizures and SD following subarachnoid hemorrhage or brain trauma, by intracerebral electrodes (Fabricius et al., 2008; Hartings et al., 2011; Dreier et al., 2012). Even though SD and seizure activity is clearly linked, a surprisingly small number of articles directly address the role of SD in the seizing brain. In this mini review we will address the role of SD as a common denominator of migraine and epilepsy, and discuss SD as a mechanism capable of terminating epileptiform activity. Moreover, we will discuss potential pathophysiological roles of reactive astrocytes in SD in the context of seizure termination. Table 1 summarizes relevant studies and reviews about co-occurrence and shared pathophysiological mechanisms between SD and epileptic activity.

**TABLE 1:** Key literature on studies and reviews underpinning co-occurrence and shared pathophysiological mechanisms between spreading depolarization and epileptic activity.

Publication	Ref.	Method	Main findings
Animal studies			
Tamin I et al., Nat Commun 2021	Tamim et al. (2021)	*In vivo* mouse model of focal neocortical seizures triggered chemically. Optogenetic or KCl-induced SDs with antiseizure effect	SD terminating epileptic activity
Heuser K et al., Cerebral Cortex 2018	Heuser et al. (2018)	2-photon calcium imaging in awake mice with epilepsy, direct observation of SD under ongoing epileptic activity	SD terminating epileptic activity
Samoteva IS et al., Neuroscience 2013	Samotaeva et al. (2013)	Intracortical microinjections of selective I_h_ channel antagonist in a genetic epilepsy model in rats	Suppression of absences seizures by SD
Human studies			
Dreier JP et al., Brain 2012	Dreier et al. (2012)	Field potential recordings of patients with aneurismal subarachnoid hemorrhage, and from neocortical slices from patients with intractable temporal lobe epilepsy	SD co-occurrence with epileptic activity
Fabricius M et al., Clin Neurophysiol 2008	Fabricius et al. (2008)	Intracortical EEG in acute brain injury	SD co-occurrence with epileptic activity
Zangaladze A et al. Epilepsy Behav, 2010	Zangaladze et al. (2010)	Phenotype-genotype correlation	Sporadic hemiplegic migraine and epilepsy associated with CACNA1A gene mutation
Berger M et al. Cephalalgia, 2008	Berger et al. (2008)	Human neocortical slice experiments	SD enhances human neocortical excitability *in vitro*
Review articles			
Dreier, JP. Nat Med 2011	Dreier (2011)	The role of spreading depression, spreading depolarization and spreading ischemia in neurological disease	
Rogawski, MA Arch Neurol 2008	Rogawski (2008)	Common pathophysiologic mechanisms in migraine and epilepsy	
Haut SR et al. Lancet Neurol 2006	Haut et al. (2006)	Chronic disorders with episodic manifestations: focus on epilepsy and migraine	
Dreier JP et al., J Cereb Blood Flow Metab 2017	Dreier et al. (2017)	Recording, analysis, and interpretation of spreading depolarizations in neurointensive care: Review and recommendations of the COSBID research group	
Bauer PR et al. Nat Rev Neurol 2021	Bauer et al. (2021)	Headache in people with epilepsy	

## Clinical overlap between migraine and epilepsy

The association between migraine and epilepsy was already recognized more than a century ago (Gowers, 1906; Noebels, 2012; Rogawski, 2012). Migraineurs exhibit a significantly elevated risk of epilepsy compared to non-migraineurs, mirroring the increased prevalence of migraine in populations with epilepsy (Ottman and Lipton, 1994; Haut et al., 2006; Bagheri et al., 2020). Both migraine and epilepsy manifest as chronic disorders with recurrent episodic attacks and interictal wellbeing (Ryan and Ptácek, 2010). In some instances, the attacks persist, leading to status epilepticus or status migrainosus, respectively. Both migraine attacks and epileptic seizures go through distinct phases, including prodromal and/or aura phases, the attack itself, and a postdromal or postictal phase.

While inducing a seizure through migraine is uncommon, headaches often accompany seizures. Although preictal and ictal headaches are infrequent, postictal headaches are prevalent and may exhibit migraine-like features (Silberstein et al., 2008). Both migraine and epilepsy may present with a range of symptoms involving visual, auditory, somatosensory, or motor features. In some cases, migraine aura may precede seizures, a phenomenon termed migralepsy (Lennox, 1960; Cianchetti et al., 2013; Headache Classification Committee of the International Headache Society IHS, 2018). While migraine boasts a broader array of triggering factors compared to epilepsy, numerous shared triggers exist, such as sleep deprivation, alcohol, bright light, stress or stress relief, and hormonal changes.

Strong support for a shared genetic basis emerges from the identification of genes implicated in both epilepsy and migraine. Phenotypic-genotypic correlations, particularly mutations in the genes CACNA1A (P/Q-type voltage-gated Ca^2+^ channel), ATP1A2 (Na^+^-K^+^ ATPase), SCN1A (voltage-gated Na^+^ channel), are observed in familial hemiplegic migraine, as well as in generalized and focal epilepsies (Chioza et al., 2001; Zangaladze et al., 2010).

Also in terms of treatment, a clear overlap exists between migraine and epilepsy. Robust evidence, including findings from randomized controlled clinical trials, supports the effectiveness of anti-seizure medications such as valproate, topiramate, gabapentin, pregabalin, levetiracetam, zonisamide, and lamotrigine as preventive drugs for migraine (Olesen and Ramadan, 2008; D’Amico, 2010; Calandre et al., 2010; Bermejo and Dorado, 2009; Villani et al., 2011; Lampl et al., 2005). Furthermore, case reports suggest that sumatriptan may be effective in treating postictal headaches (Jacob et al., 1996). The extensive overlap across clinical presentation, genetic predisposition and therapeutic response is suggestive for a common pathophysiological framework.

An elegant way to study the spatiotemporal co-occurrence of SD and epileptic activity is by intracranial electrocorticography (ECoG). In neurocritical care, this method may be an important future tool for prognostication and personalized treatment (Dreier et al., 2017). In human ECoG recordings, SD are easily distinguishable from ictal activity because the negative DC shift of SD is several times larger than the negative DC shift of an ictal event, and the propagation rate is usually much faster in the latter (Dreier et al., 2012). Both events are often co-occurring in acute brain conditions, including in patients with acute status epilepticus (Fabricius et al., 2008). However, SD in human epilepsy is understudied. Whether SD has a role in epileptogenesis, defined as the transformation of normal brain matter to one that is prone to generate epileptic activity, remains to be investigated. SD facilitates neuronal death, which is one key feature of epileptogenesis. Notably, early SD showed a significant association with the development of late epilepsy in patients with acute subarachnoid hemorrhage (Dreier et al., 2012). A recent small prospective study performed in patients with serious traumatic brain injury and malignant ischemic stroke found no association between SD events and epileptogenesis (Sueiras et al., 2021).

## Spreading depolarization waves as local emergency brakes

The brain operates on a fine balance between excitation and inhibition, and potentially, only small relative reductions in inhibition or increases in excitation may cause synchronization of neural networks and resultant seizure activity (Isaacson and Scanziani, 2011). Generalized seizures, particularly in an evolutionary perspective, are detrimental as they render the organism incapable of reacting to external stimuli and evading predation, in addition to potential injuries from falling, or death. Hence, potent mechanisms should be in place to prevent seizure activity. Since SDs are known to co-occur with seizures, can be elicited by some of the same mechanisms as seizures, and strongly suppress neuronal activity for minutes, it is tempting to speculate that SD could be one such mechanism.

The exact mechanistic underpinnings underlying SD initiation and propagation are not known. However, it is believed that increases in extracellular K^+^ concentration, and potentially an interplay with extracellular glutamate, are key events, setting in motion a self-regenerating wave-like depolarization. Triggering of SD experimentally can be achieved in many ways, but likely a common denominator of all these methods is an increase of K^+^ above a threshold level of ∼15 mM in a sufficiently large extracellular volume (Tang et al., 2014; Wei et al., 2014), an increase of local extracellular glutamate (Parker et al., 2021), or an interplay between these two factors. For the propagation of SD, we have demonstrated that elevations in extracellular K^+^ seems to precede any other significant local cellular event by several seconds (Enger et al., 2015). This is in line with one of the earliest hypotheses about SD, namely that spread of extracellular K^+^ released by depolarized neurons serves to depolarize neighboring cells, leading to more release of K^+^ and a self-regenerating wave of depolarization (Grafstein, 1956). The exact mechanisms are, however, still unclear.

**FIGURE 1:**
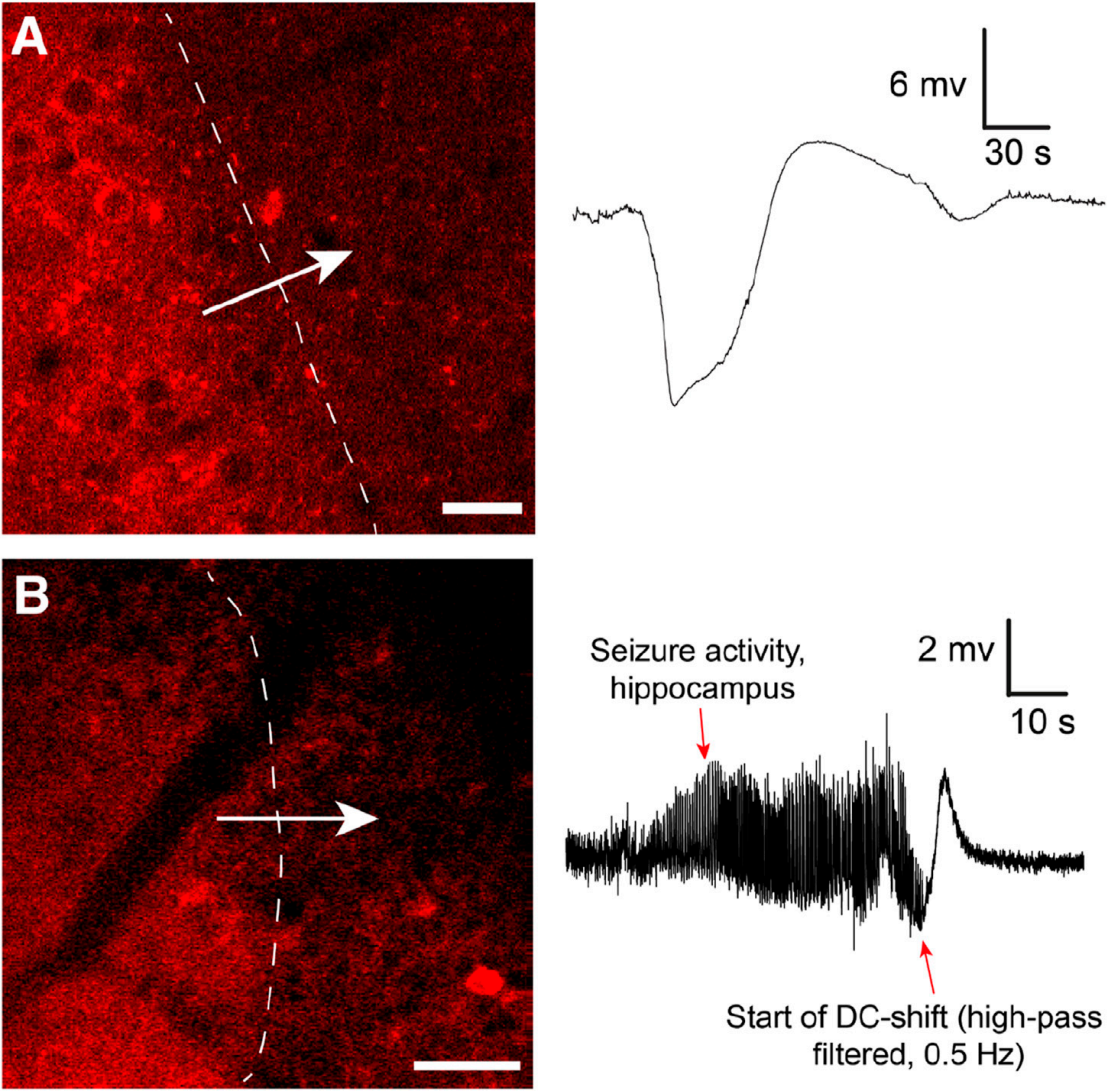
**(A)** Cortical spreading depression wave induced by KCl imaged by *in vivo* two-photon microscopy using the genetically encoded fluorescent sensor jRCaMP1a in unanesthetized mice. The sensor was expressed under the human *synapsin1* promoter. In the awake unanesthetized state these waves move with a speed of 4.5–6 mm/min. Note typical shape of recorded DC shift. Adapted from Enger et al., 2017 Cerebral Cortex (Enger et al., 2017). Scale bar 25 μm. **(B)** Similar waves were imaged during Kainate-induced seizures in the hippocampus. In this experiment neuronal Ca^2+^ dynamics were imaged with jRGECO1a under the human *synapsin1* promoter. To record seizure activity the hippocampal local field potential electrode signal had to be high-pass filtered to compensate for signal drift and saturation of signal. This distorts the classical DC shift, as also reported in (Dreier et al., 2017). Adapted from Heuser et al., 2018 Cerebral Cortex (Heuser et al., 2018). Scale bar 50 μm.

The changes associated with SD in the brain tissue are dramatic, and it has been clearly shown that SD waves are detrimental to already compromised brain tissue (Lauritzen et al., 2011). Hence, most studies on SD currently focus on the damaging effects of SD. However, the role of SD in migraine may suggest that given an uncompromised metabolic supply, SD may not be such a detrimental event (Hadjikhani et al., 2001; Lauritzen et al., 2011). After all, migraineurs may experience hundreds of auras throughout a lifetime with strikingly few realized long-term consequences. Our data, directly visualizing SD in cortex in awake behaving mice with two-photon microscopy (Enger et al., 2017) (Figure 1), corroborate this idea as we find that in the awake state with unperturbed physiological conditions, brain tissue regenerates from SD events considerably faster than what has been reported in anesthetized mice (Enger et al., 2017). SDs are relatively frequent occurrences in the population. For instance, the lifetime prevalence of migraine has been estimated to be ∼30%, of which about a quarter experience aura symptoms (Russell and Olesen, 1996; Kim et al., 2022). Recordings in humans suggest that SDs outside primary sensory areas are difficult to detect for the patient, or give subtle unusual symptoms pertaining to association cortices (Bowyer et al., 2001; Vincent and Hadjikhani, 2007; Hadjikhani and Vincent, 2021). Hence, it could be that SD are more frequent than what patients report in the form of symptomatic migraine auras (Hadjikhani and Vincent, 2021).

Little focus has been given to the potential beneficial roles of SD, and why this seemingly pathological event is so very well conserved through phylogeny (Ayata and Lauritzen, 2015). SD can be induced in a broad range of species, from grasshoppers and mudpuppies to man (Pietrobon and Moskowitz, 2014; Ayata and Lauritzen, 2015; Kramer et al., 2016). Because of this ability of the nervous tissue to sustain such activity, and its apparent prevalence at least in humans, it is tempting to speculate that SD may serve beneficial roles. In the aftermath of spreading depolarizations the neurons are silenced for several minutes, and potentially curbing brain hyperexcitation is a beneficial physiological role for SD. SDs may serve as an emergency brake that prevent localized hyperexcitation to transform into a full-blown seizure, and potentially SD plays a role in ending seizure activity in the brain.

## Spreading depolarization silence neuronal activity during seizures

The spontaneous termination of seizures remains a fundamental question in the field of epileptology without a definitive answer (Lado and Moshé, 2008; Löscher and Köhling, 2010). As proposed by Kramer et al. brain seizure activity undergoes a critical transition when approaching termination, that can be observed in EEG (Kramer et al., 2012). Especially prolonged seizures, such as those seen in status epilepticus, repeatedly approach but do not surpass this critical transition (Kramer et al., 2012). Several mechanisms have been proposed to play a role in seizure termination, including increased GABAergic signaling, neurotransmitter or ATP depletion, ionic imbalance, or release of adenosine, which has an inhibitory effect on neurons (Lado and Moshé, 2008; Kramer et al., 2012). Could it be that SD in the seizure generating circuitry plays a role in seizure termination?

A phenomenon sharing both clinical and electrophysiological features with SD is postictal depression (PD). PD refers to the period of altered brain activity that follows a seizure (So and Blume, 2010). Electrophysiologically it is characterized by suppression and neuronal activity similar to what is observed in SD. PD is defined as abnormal slow-wave activity or EGG amplitudes of <10 µV within 30 s of seizure cessation, lasting more than 2 s (So and Blume, 2010; Bateman et al., 2019). It has been found in 84% of seizures and in 94% of epilepsy patients (Bateman et al., 2019). Key clinical features of PD are mood changes, fatigue, cognitive impairment, and also physical symptoms like headache and muscle aches, all phenomena also observed in migraine patients (Pottkämper et al., 2020). While the exact mechanisms of postictal depression are not understood, researchers have proposed several factors that may contribute (Bruno and Richardson, 2020; Pottkämper et al., 2020). Although PD often manifests as a generalized phenomenon, which is difficult to reconcile with the relatively localized nature of SD, SDs in key parts of the seizure circuitry, like the hippocampus, may play a role.

Few investigators have looked into the actual role of SD in seizure termination. In Heuser et al. 2018, we showed that kainate-induced seizure activity in the hippocampus appeared to be terminated by SD waves (Heuser et al., 2018) (Figure 1): After SD, no epileptic activity was recorded locally for several minutes, before seizure activity typically re-emerged. Similar observations were made in a study presenting a model for inducing seizures by optogenetically stimulating cortical interneurons, where they observed fluorescence waves characteristic of SD waves (Khoshkhoo et al., 2017). In Samotaeva et al. 2013 they observed that SD induced by cortical sham microinjections suppressed spike-wave discharges in WAG/Rij rats, a genetic model for absence epilepsy, for up to 90 min (Samotaeva et al., 2013). In another study in the same rat model that also displays audiogenic seizures, they found that audiogenic seizures associated with SD were associated with a suppression of spike-wave discharges for over 1 h (Vinogradova et al., 2005). Recently, in Tamim et al. 2021 they elegantly demonstrated that SD in addition to often occuring during seizure activity also played a prominent role in terminating seizure activity, and even prevented generalization of seizures (Tamim et al., 2021). They showed that focal seizures elicited by 4-aminopyridine, penicillin and bicuculline all triggered SD events, in particular if the seizure activity spread widely. Moreover, by inducing SD experimentally during seizure activity they were able to both prevent seizure generalization and terminate seizure activity (Tamim et al., 2021). In the same study they also showed that inhibiting SDs pharmacologically led to more severe seizure activity and a higher chance of generalization of seizure activity. In another study using Kv1.1 potassium channel knockout mice or mice with a knock-in mutation in the *Scn1a* gene known to cause Dravet Syndrome in humans, brainstem SD were found during induced seizure activity (Aiba and Noebels, 2015).

These studies were performed in acute seizure models, or in induced seizures in seizure susceptible animal models. Since seizures and SD can be triggered by many of the same agents, another question is to what extent SDs also occur in relation to spontaneous seizures in chronic seizure models. A recent preprint provides evidence suggesting that indeed SD is a hallmark of chronic epilepsy models as well (Bahari et al., 2020). They demonstrate that in a chronic tetanus toxin model of temporal lobe epilepsy in rats, and in a post cerebral malaria model of epilepsy around one-third of spontaneous seizures were associated with SD (Bahari et al., 2020). Data from humans demonstrating SDs co-occurring with seizures are scarce, likely because standard scalp EEG does not pick up SDs (Dreier et al., 2017). Moreover, intracerebral recordings are often slightly high pass-filtered obscuring the classical direct current shifts associated with SD (Dreier et al., 2017). A handful of studies have demonstrated co-occurring seizures and SD following subarachnoid hemorrhage or brain trauma by intracerebral electrodes (Fabricius et al., 2008; Hartings et al., 2011; Dreier et al., 2012). In these studies typically SDs are recorded in a subset of seizures. However, since SDs are relatively localized events, it could be that the reported rates are underestimating the true occurrence.

Complicating the interpretation of SD in curbing hyperexcitation is that experimental data suggest that neurons both locally and in other parts of the brain are hyperexcitable in the aftermath of a SD wave, even 45 min after the event (Wernsmann et al., 2006; Berger et al., 2008; Ghadiri et al., 2012). Potentially these findings could explain hyperexcitation symptoms in migraineurs like photophobia. In Bahari et al. 2020 they also reported that the interval between seizures for seizure bouts associated with SD was shorter than for seizures without SD (Bahari et al., 2020). However, that does not necessarily mean that SD promotes seizures: In Tamim et al. 2021, Samotaeva et al. 2013 and Vinogradova et al. 2005, a clear suppression in epileptiform activity for 30–90 min following SD was observed (Samotaeva et al., 2013).

Another intriguing aspect is that many pharmacological agents, including anti-seizure medication, have been shown to impede SD (Klass et al., 2018). However, these drugs also attenuate excitability in general and by this also target SD. The differential effects on pharmacological agents on epileptiform activity versus SD is to the best of our knowledge not investigated.

## Possible role of spreading depolarization in preventing generalization of seizure activity

We have here hypothesized and presented data from reports suggesting that SD may be a mechanism for the brain to curb epileptiform activity. Could SD also play a role in preventing spread of localized spontaneously occurring interictal epileptiform activity before it spreads to larger neuronal networks and becomes a clinical seizure?

A range of brain lesions and brain disorders can trigger epilepsy (Balestrini et al., 2021). Examples include structural changes associated with stroke and traumatic brain injury. A common denominator of many of these insults is that they are associated with reactive astrogliosis (as reviewed in (Pekny and Pekna, 2016; Patel et al., 2019; Escartin et al., 2021)). Reactive astrocytes are characterized by morphological, expressional and functional changes, and can exist in mild reversible forms, or in the extreme case, as glial scar tissue (Escartin et al., 2021). There are multiple lines of evidence suggesting that reactive astrogliosis could be pro-epileptic, although the precise mechanisms are unknown (Robel et al., 2015; Zhu et al., 2016; Patel et al., 2019; Sano et al., 2019; Heuser et al., 2021; Çarçak et al., 2023). An illustrative example of reactive gliosis in epilepsy is mesial temporal lobe epilepsy with progressive gliotic transformation of the hippocampi (Bedner et al., 2015; Walker, 2015; Balestrini et al., 2021). This disease entity is relatively well characterized in terms of the histopathological changes in the tissue. Interestingly, investigations of resectates from patients undergoing surgery for MTLE have shown a range of molecular changes in reactive astrocytes, including loss of the potassium channel Kir4.1 (Heuser et al., 2012), changes in gap junction subtypes (Fonseca et al., 2002) and loss of gap junctional coupling (Bedner et al., 2015), loss of glutamine synthetase (Eid et al., 2004) and aquaporin-4 (Lee et al., 2004). In preclinical studies a range of other mechanisms, including astrocyte signaling has been implicated (Shigetomi et al., 2019; Heuser and Enger, 2021). The exact mechanisms by which the molecular and morphological changes in reactive astrocytes affect propensity to develop epileptiform activity is unknown. Likely some of the changes observed could also be protective, hindering hyperexcitation.

If one takes the perspective that SD may have beneficial effects in hyperexcitation disorders by acting as emergency brakes for excitation, one could also conjecture that a higher threshold for SD would be pro-epileptogenic. Astrocytes and astrocytic molecules are known to affect SD propagation and initiation (Seidel et al., 2016; Enger et al., 2017). Importantly, astrocytes are crucial for clearance of extracellular K^+^ and glutamate, as discussed above in the context of reactive astrogliosis, which are key mechanisms involved in SD initiation and propagation. One illustrative example is familial hemiplegic migraine caused by mutations in the ATP1A2 gene that encodes the predominant Na^+^-K^+^ ATPase in astrocytes (Gritz and Radcliffe, 2013).

Interestingly, recent work demonstrates that reactive astrogliosis increases the threshold for SD (Seidel et al., 2015; Seidel et al., 2016). Here they used lentiviral transfection of neurons so that they constitutively express ciliary neurotrophic factor, a cytokine that induces a reactive astrocyte phenotype (Escartin et al., 2006; Escartin et al., 2007). Using an *in vitro* model for SD by application of K^+^ on acute brain slices they observed a much higher threshold for eliciting SD in slices with reactive astrocytes, despite otherwise normal neuronal excitability. Potentially this effect was mediated through augmented astrocytic K^+^ uptake mechanisms in the reactive astrocytes. Another *in vivo* study clearly demonstrates that SD susceptibility is lower after repeated SD induction which leads to a reactive astrogliosis. However, they did not establish a causal relationship (Sukhotinsky et al., 2011). These are interesting observations, but more research is needed to establish whether attenuated SD susceptibility in reactive astrogliosis also occurs in epilepsy, and whether such mechanisms contribute to seizure propensity.

## Conclusion

The brain operates on a fine balance between excitation and inhibition, and potent mechanisms of curbing hyperexcitation are essential for survival. Accruing evidence indicates that SDs frequently occur in seizures, and potentially have a role in curbing hyperexcitation. If SD has such a role, perturbed SD mechanisms in reactive astrogliosis could potentially contribute to seizure propensity in epilepsy. Further studies are needed to elucidate the role of SD in epilepsy, and to leverage this potential anti-seizure mechanism to treat epilepsy.
